# A medium-chain triglyceride containing ketogenic diet exacerbates cardiomyopathy in a CRISPR/Cas9 gene-edited rat model with Duchenne muscular dystrophy

**DOI:** 10.1038/s41598-022-15934-9

**Published:** 2022-07-08

**Authors:** Yuri Fujikura, Koichi Kimura, Keitaro Yamanouchi, Hidetoshi Sugihara, Masaki Hatakeyama, Haotong Zhuang, Tomoki Abe, Masao Daimon, Hiroyuki Morita, Issei Komuro, Katsutaka Oishi

**Affiliations:** 1 Healthy Food Science Research Group, Cellular and Molecular Biotechnology Research Institute, National Institute of Advanced Industrial Science and Technology (AIST), Tsukuba, Ibaraki 305-8566 Japan; 2 Departments of Laboratory Medicine / Cardiology, The Institute of Medical Science, The University of Tokyo, Minato-ku, Tokyo 108-8639 Japan; 3 Department of Veterinary Physiology, Graduate School of Agricultural and Life Sciences, The University of Tokyo, Bunkyo-ku, Tokyo 113-8657 Japan; 4 Miyagi Health Innovation, Sendai, Miyagi 980-0021 Japan; 5 Department of Cardiovascular Medicine, Graduate School of Medicine, The University of Tokyo, Bunkyo-ku, Tokyo 113-8655 Japan; 6 Department of Applied Biological Science, Graduate School of Science and Technology, Tokyo University of Science, Noda, Chiba Japan; 7 Department of Computational Biology and Medical Sciences, Graduate School of Frontier Sciences, The University of Tokyo, Kashiwa, Chiba Japan; 8 School of Integrative and Global Majors (SIGMA), University of Tsukuba, Tsukuba, Ibaraki Japan

**Keywords:** Cardiomyopathies, Neuromuscular disease

## Abstract

Duchenne muscular dystrophy (DMD) is an X-linked recessive myopathy caused by dystrophin mutations. Although respiratory management has improved the prognosis of patients with DMD, inevitable progressive cardiomyopathy is a current leading cause of premature death. Recently, we showed that a medium-chain triglyceride containing ketogenic diet (MCTKD) improves skeletal muscle function and pathology in a CRISPR/Cas9 gene-edited rat model with DMD. In this study, we sought to clarify whether MCTKD also improves the cardiomyopathy in these rats. DMD rats were fed either the MCTKD or normal diet (ND) from ages of 3 weeks to 9 months old. Compared with the ND-fed rats, MCTKD-fed rats showed significantly prolonged QRS duration, decreased left ventricular fractional shortening, an increased heart weight/body weight ratio, and progression of cardiac fibrosis. In contrast to our previous study which found that MCTKD improved skeletal myopathy, the current study showed unexpected exacerbation of the cardiomyopathy. Further studies are needed to explore the underlying mechanisms for these differences and to explore modified dietary options that improve skeletal and cardiac muscles simultaneously.

## Introduction

Duchenne muscular dystrophy (DMD) is a progressive myopathy caused by mutations in the dystrophin gene on the X chromosome. Dystrophin is a component of the dystrophin-glycoprotein complex, which connects the cytoskeleton to the basement membrane^1^. The loss of dystrophin causes persistent muscle degeneration, necrosis, fibrosis, and adipose tissue replacements^2^. Consequently, patients with DMD suffer from disabilities due to progressive skeletal muscle weakness and cardiomyopathy. Although therapeutic advances including corticosteroids^3^, angiotensin converting enzyme inhibitors^4,5^ beta-blockers^6,7^, and artificial respiratory management have improved the prognosis of patients with DMD, inevitable progressive cardiomyopathy is a current leading cause of premature death^8,9^.

To develop effective therapeutic options, several animal models of DMD have been generated. The most commonly used animal model is mdx mice, which have an out-of-frame mutation in the exon 23 of *DMD*. However, the phenotype of mdx mice is mild and skeletal muscle damage and cardiomyopathy are not as severe as in human patients^10^. In recent years, we have generated a rat model of DMD (DMD rats) using a CRISPR/Cas9 genome editing system. Our previous research showed that DMD rats exhibited more severe skeletal muscle damage and cardiomyopathy phenotypes than mdx mice^11–14^. In DMD rats, cardiac degeneration and fibrosis progress with age and overt left ventricular (LV) dysfunction occurs around 8–10 months old^13,14^.

Ketogenic diets contain high fat, moderate protein, and low carbohydrate levels, which promotes the production of internal ketone bodies (Supplemental Fig. 1A)^15^. The traditional ketogenic diet is known to have beneficial effects for many conditions such as intractable epilepsy^16^, Alzheimer’s disease^17^, and Parkinson’s disease^18^. However, the traditional ketogenic diet mainly contains long-chain triglycerides (LCT) for the fat components, and LCT containing ketogenic diets (LCTKD) have limited efficiency in the process of converting fat components to ketone bodies. As a result, the relatively low ratios of carbohydrates and protein in LCTKD could cause risks for muscle catabolism^15^ and atrophy^19,20^. To overcome these risks, medium-chain triglycerides (MCT) were developed from the seeds of palm plants as an alternative choice to the traditional LCT (Supplemental Fig. 1B)^21^. Unlike LCT, MCT can directly flow into the portal vein and can rapidly be converted to ketone bodies in the liver. Therefore, an MCT containing ketogenic diet (MCTKD) raises blood ketone levels more efficiently than LCTKD and may contain sufficient protein and carbohydrate for muscle metabolism.

We have recently reported that MCTKD feeding intervention improves skeletal muscle function and pathology in DMD rats^22^. The skeletal muscle necrosis, inflammation, and fibrosis were significantly suppressed in these rats. In addition, MCTKD-fed DMD rats showed enhanced muscle regeneration via the promotion of proliferation of satellite cells. These results suggest that MCTKD can be a novel dietary treatment option for patients with DMD. The purpose of this study was to evaluate whether MCTKD dietary intervention also has a beneficial effect on DMD cardiomyopathy.

## Results

### Electrocardiogram evaluation

**Figure 1:**
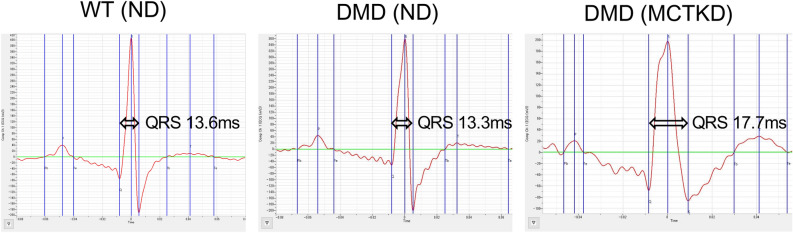
Representative electrocardiogram images. A significant prolongation of QRS duration was observed in MCTKD-fed DMD rats. DMD = Duchenne muscular dystrophy, MCTKD = medium-chain triglycerides containing ketogenic diet, ND = normal diet, WT = wild type.

Electrocardiograms were successfully evaluated in all animals. In the comparison between ND-fed DMD rats and MCTKD-fed DMD rats, there were no significant differences in heart rate, P-wave amplitude, PR interval, or QT interval (Table 1). The R-wave amplitude in MCTKD-fed DMD rats was significantly lower than that in ND-fed DMD rats. Also, QRS duration in the MCTKD-fed rats was significantly longer than that in the ND-fed DMD rats (Fig. 1).

**Table 1:** Electrocardiogram results.

	WT (ND)	DMD (ND)	DMD (MCTKD)	*P* value
Heart rate (bpm)	356 ± 17	274 ± 16	270 ± 11	0.978
P-wave amplitude (mV)	45.8 ± 7.6	35.4 ± 3.5	28.8 ± 5.0	0.608
R-wave amplitude (mV)	359 ± 33	371 ± 29	248 ± 17	0.010
PR interval (ms)	50.4 ± 5.7	47.1 ± 4.0	47.9 ± 4.3	0.991
QRS duration (ms)	15.7 ± 0.8	14.2 ± 0.6	16.7 ± 0.5	0.021
QT interval (ms)	71.5 ± 2.2	63.0 ± 2.3	62.3 ± 1.7	0.967

Values are the mean ± SEM (n = 6 for ND-fed DMD rats, n = 6 for MCTKD-fed DMD rats, n = 4 for WT rats). *P* values indicate the results of one-way ANOVA followed by the Tukey–Kramer multiple comparison test between 2 groups of DMD (ND) and DMD (KD). DMD = Duchenne muscular dystrophy, MCTKD = medium-chain triglycerides containing ketogenic diet, ND = normal diet, WT = wild type.

### Echocardiography evaluation

**Figure 2:**
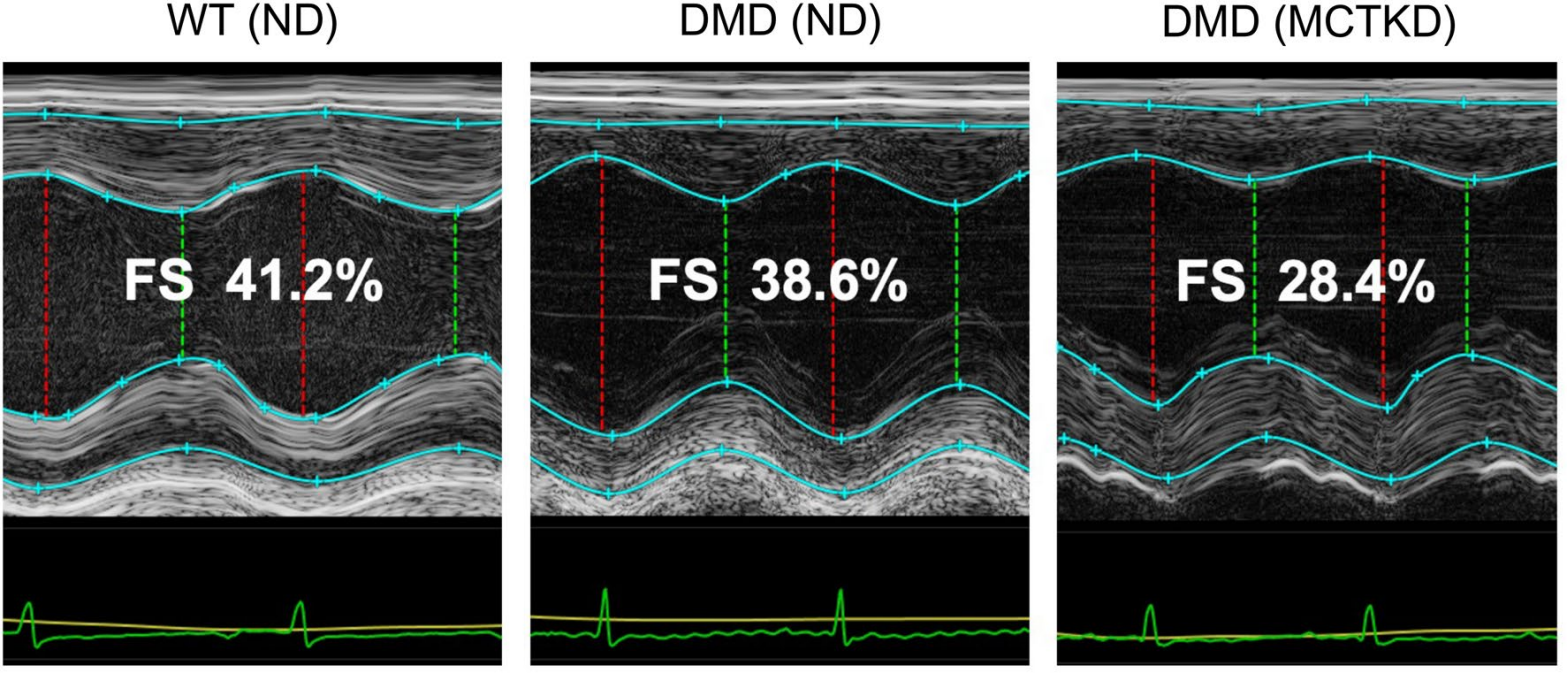
Representative images of echocardiography. A significant decrease in left ventricular (LV) fractional shortening (FS) was observed in MCTKD-fed DMD rats. Red and green lines indicate LV diameters at end-diastole and end-systole, respectively.

**Figure 3:**
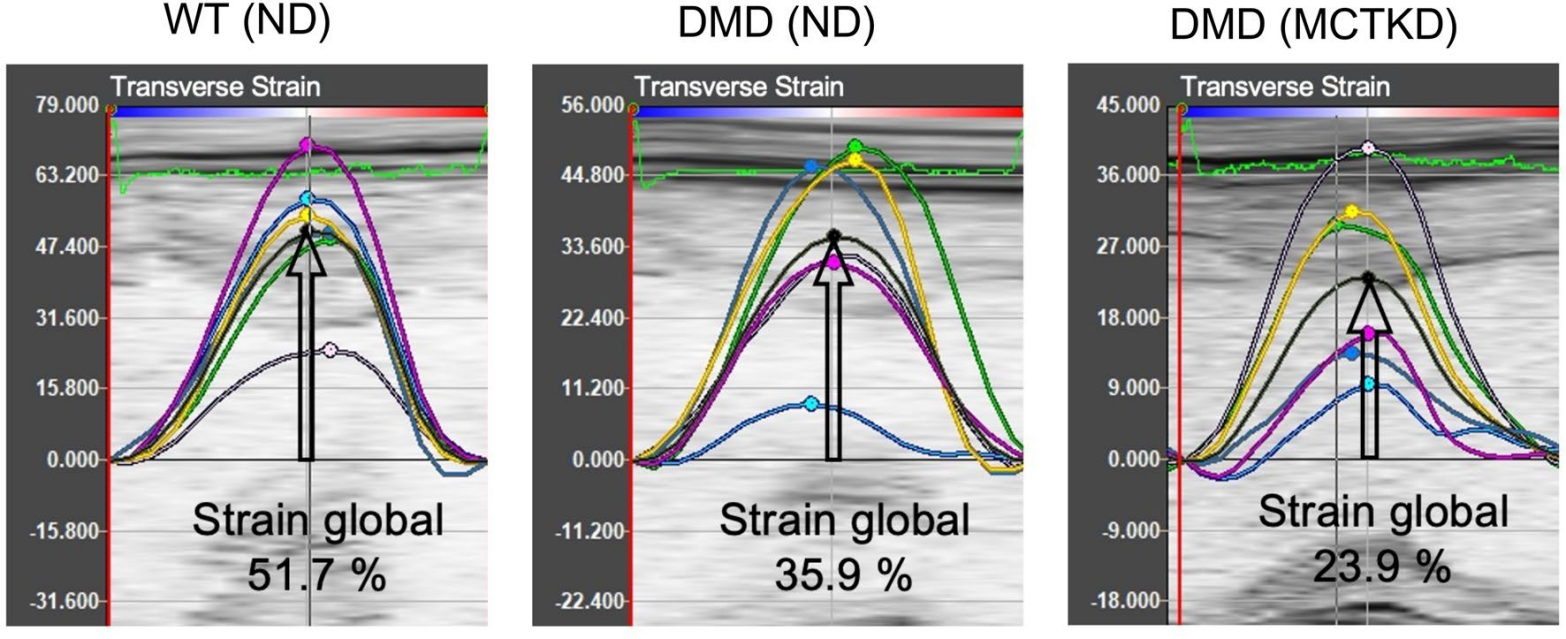
Representative images of speckle tracking strain analysis. A trend toward a decrease in transverse strain global was observed in MCTKD-fed DMD rats (arrows). The colored curves indicate the strain of each segment. The black curve indicates strain global of all segments.

Echocardiography imaging was successfully evaluated in all animals. ND-fed DMD rats showed decreased LV systolic and diastolic function in comparison to the ND-fed WT rats. Significant differences were observed in the strain rate variables; Radial strain rate S-peak (*P* = 0.048), longitudinal strain rate E-peak (*P* = 0.002), and transverse strain rate E-peak (*P* = 0.004). Furthermore, the MCTKD-fed DMD rats showed a significant decrease in LV fractional shortening (*P* = 0.011) in comparison to the ND-fed DMD rats (Fig. 2). Although the differences were not statistically significant due to the limited number of animals, most systolic and diastolic variables (Table 2) exhibited a trend toward worsening in the MCTKD-fed DMD rats compared to the ND-fed DMD rats (Fig. 3).

**Table.2:** Echocardiography results.

	WT (ND)	DMD (ND)	DMD (MCTKD)	*P* value
**Conventional variables**				
LV fractional shortening (%)	41.5 ± 0.8	36.3 ± 1.6	29.0 ± 1.8	0.011
LV end-diastolic anterior wall thickness (mm)	1.6 ± 0.1	1.4 ± 0.1	1.6 ± 0.1	0.606
LV end-diastolic posterior wall thickness (mm)	2.8 ± 0.1	2.0 ± 0.2	2.3 ± 0.1	0.215
LV end-diastolic diameter (mm)	9.0 ± 0.3	8.4 ± 0.2	8.5 ± 0.4	0.943
LV ejection fraction (%)	59.7 ± 2.0	57.7 ± 3.6	49.4 ± 4.1	0.247
**Strain analysis (systolic function)**				
Circumferential strain global (%)	26.4 ± 1.5	21.0 ± 1.9	20.5 ± 3.1	0.990
Radial strain global (%)	40.9 ± 5.1	27.0 ± 3.9	29.3 ± 5.0	0.935
Longitudinal strain global (%)	21.2 ± 1.4	16.2 ± 2.4	10.6 ± 1.5	0.114
Transverse strain global (%)	47.2 ± 2.1	36.9 ± 7.4	25.2 ± 3.6	0.277
Circumferential strain rate S-peak (/s)	5.6 ± 0.4	4.0 ± 0.4	3.5 ± 0.5	0.633
Radial strain rate S-peak (/s)	5.7 ± 0.4	3.9 ± 0.4	4.1 ± 0.5	0.898
Longitudinal strain rate S-peak (/s)	4.4 ± 0.4	3.3 ± 0.8	2.2 ± 0.3	0.359
Transverse strain rate S-peak (/s)	6.8 ± 0.1	4.9 ± 0.9	3.6 ± 0.4	0.290
**Strain analysis (diastolic function)**				
Circumferential strain rate E-peak (/s)	6.7 ± 0.7	5.2 ± 0.8	4.5 ± 1.0	0.808
Radial strain rate E-peak (/s)	6.6 ± 1.0	5.1 ± 0.7	4.9 ± 1.0	0.980
Longitudinal strain rate E-peak (/s)	6.5 ± 0.9	3.2 ± 0.4	1.8 ± 0.3	0.114
Transverse strain rate E-peak (/s)	9.2 ± 0.9	5.0 ± 0.7	3.6 ± 0.6	0.331

Values are the mean ± SEM (n = 6 for ND-fed DMD rats, n = 6 for MCTKD-fed DMD rats, n = 4 for WT rats). *P* value indicates the results of one-way ANOVA followed by the Tukey–Kramer multiple comparison test between 2 groups of DMD (ND) and DMD (KD). E-peak = peak strain rate at early diastole, LV = Left ventricular, S-peak = peak strain rate at systole.

### Cardiac histopathology

**Figure 4:**
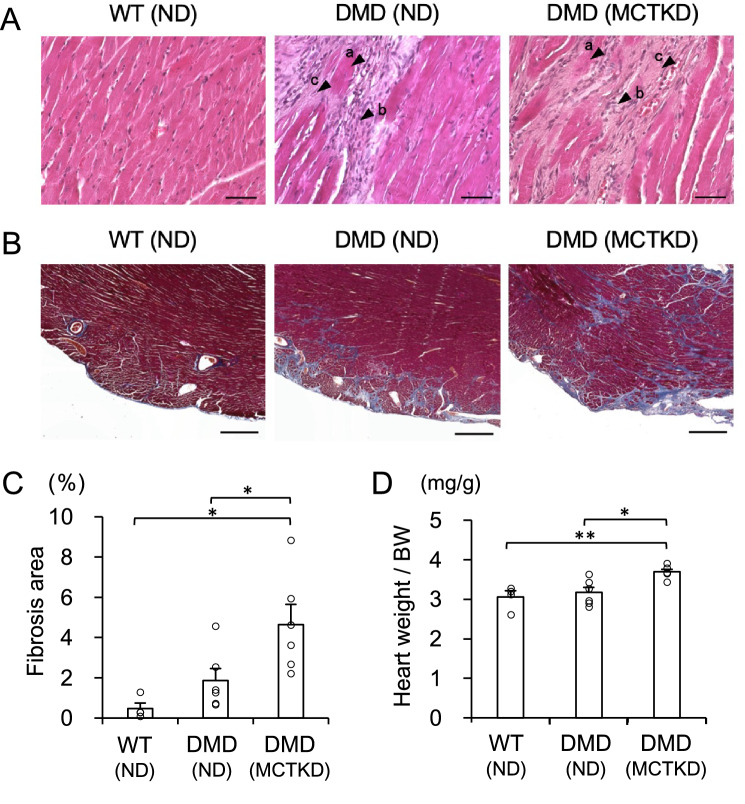
Histopathological analyses of left ventricular tissues. (**A**) Haematoxylin–eosin-stained sections showed necrosis (a), inflammatory cell infiltration (b) and fibrosis (c) in DMD rats. Scale bars indicate 50 μm. (**B**) Representative Masson’s trichrome stained sections showed significant fibrosis in DMD rats. Scale bars indicate 500 μm. (**C**) The ratio of quantified fibrotic area was significantly increased in MCTKD-fed DMD rats compared to that in ND-fed DMD rats (*P < 0.05). (**D**) The ratio of whole heart weight divided by body weight (BW) in MCTKD-fed DMD rats was significantly greater than in ND-fed DMD rats (*P < 0.05). Bar graphs (**C**-**D**) are expressed as the mean ± SEM and the values were compared by one-way ANOVA followed by the Tukey–Kramer multiple comparisons test (n = 6 for ND-fed DMD rats, n = 6 for MCTKD-fed DMD rats, n = 4 for WT rats).

Histopathological analysis (Fig. 4A) revealed cardiomyocyte necrosis, infiltration of mononuclear cells, and fibrosis in the DMD rats, while they were not observed in the WT rats. Although the immunostaining of IgG (Supplemental Fig. 3) did not show a significant difference, Masson's trichrome staining (Fig. 4B) showed a more significant increase in the percentage of fibrotic area (Fig. 4C) in the MCTKD-fed DMD rats (4.7 ± 1.0%) as compared with the ND-fed DMD rats (1.9 ± 0.6%, *P* = 0.046). The whole heart weight ratios to body weight (Fig. 4D) in the MCTKD-fed DMD rats (3.6 ± 0.1 mg/g) were significantly increased compared to those in the ND-fed DMD rats (3.1 ± 0.1 mg/g, *P* = 0.011).

## Discussion

We expected to observe the beneficial effects of MCTKD dietary intervention in cardiac muscle since improvements in skeletal muscle were previously reported^22^. However, we observed the unexpected negative finding that MCTKD feeding did not ameliorate cardiomyopathy in DMD rats, but rather exacerbated the LV dysfunction and fibrosis. To the best of our knowledge, this study is the first report regarding the effects on cardiac function and pathology by MCTKD intervention.

Several papers have reported on the beneficial effects of a traditional ketogenic diet, LCTKD, on cardiac function. In mouse models, ketogenic diets showed cardio-protective effects in aortic constriction^23^ and myocardial remodeling^24^ experiments. In rat models, ketogenic diets showed cardio-protective effects in hypertensive heart disease^25–27^. Even in patients with heart failure and reduced ejection fraction, the infusion of 3-hydroxybutyrate, which is a ketone body that increases plasma 3-hydroxybutyrate, showed beneficial hemodynamic effects^28^.

In regard to systemic muscle diseases, a lack of protein and carbohydrates in metabolism promotes muscle atrophy^19,20^. The traditional ketogenic diet has limited efficiency in converting LCT to ketone bodies^15^, which results in limitations in the ratio of the protein and carbohydrate concentrations to lipid concentration. From this metabolic point of view, the traditional ketogenic diet LCTKD may not be suitable for muscle diseases. On the other hand, MCT reduces the ratio of lipids and increases the ratios of carbohydrates and proteins, which results in more efficient ketone body production than LCT^15^ in muscle metabolism. Indeed, MCTKD showed a beneficial impact on skeletal muscle in DMD rats, as we expected^22^. However, MCTKD did not ameliorate cardiomyopathy. This discrepancy may have arisen for 3 possible reasons; 1) metabolic differences, 2) exercise load, and 3) regenerative capacity.

Metabolic differences between cardiac and skeletal muscle tissues have been reported. In the skeletal muscle of DMD, transition to slow-type fibers occurs and as the number of slow-type fibers increases, mitochondrial oxidative phosphorylation is compensatory increased to metabolize fat components^29^. In the cardiomyopathy of DMD, energy substrates switch from preferentially using fatty acids to glucose oxidation^30,31^. This metabolic remodeling might be due to the activation of AMPK in skeletal muscle^29^ and the suppression of PPARs in cardiomyocytes^31^. Because MCTKD is a high-fat and low-carbohydrate diet, DMD skeletal muscles are efficiently supplied with energy, although the DMD cardiac muscles, which depend on the glycolytic system, may have been suffering from energy deficiency.

Excessive exercise load can exacerbate the cardiomyopathy of DMD. In the mdx mice model, treadmill exercise was shown to worsen cardiac function and fibrosis^32,33^. Also, it was observed that patients with Becker muscular dystrophy present with severe LV dysfunction with mild or no skeletal muscle symptoms^34^. This suggests that patients with mild skeletal muscle symptoms can exercise, which increases their cardiac workload, resulting in exacerbated cardiomyopathy. In this study, the improvement of skeletal muscle function in the MCTKD-fed DMD rats may have increased their physical activity and cardiac load, which may have in turn promoted cardiac fibrosis and cardiac dysfunction.

Another possible cause is the difference in the regenerative capacity of skeletal and cardiac muscles. Although injured skeletal muscle can be quickly regenerated by stem cells, the regenerative capacity of adult mammalian cardiomyocytes is limited^35^. Therefore, the regeneration-promoting effect of MCTKD^22^ was not as effective in cardiac muscle as in skeletal muscle, leading to inter-tissue differences in the pathological changes.

From a future perspective of improving both skeletal and cardiac muscles simultaneously, modification of the nutritional components of MCTKD could be an alternative option. According to recent studies, the intake of omega-3 polyunsaturated fatty acids (PUFA)^36^ and a decreased ratio of omega-6 to omega-3 PUFA^37,38^ could ameliorate myocardial fibrosis and heart failure. MCTKD in the present study contained a large amount of unsaturated fatty acids, mostly omega-6 PUFA, with little omega-3 PUFA. Dietary intake of omega-3 PUFA was shown to improve the skeletal and cardiac muscles of mdx mice by reducing inflammation and promoting muscle regeneration^39^. Therefore, the modification of MCTKD fat components, in which some of the omega-6 PUFA are replaced with omega-3 PUFA, is expected to be an effective dietary treatment option for DMD skeletal muscle myopathy as well as the cardiomyopathy.

### Limitations

Because the number of animals to be examined was limited, many variables did not reach statistical significance. In human clinical echocardiography, Simpson’s method, the longitudinal strain/strain rate, and transverse strain/strain rate are usually analysed using the apical long-axis approach. However, in this study, we used the parasternal long-axis view to analyse these variables, because of the limited image quality of the animal apical long-axis view^40,41^. Moreover, the ratio of frame rates to the animal heart rates was limited for obtaining adequate 2D echocardiographic results of traced EF and strain variables. Therefore, the results for these variables in the present animal experiments may not be suitable to compare with the values in patients in clinical settings.

## Conclusion

The present findings demonstrated that MCTKD dietary intervention in DMD rats, which dramatically improved the skeletal muscle myopathy, did not improve their cardiomyopathy. Further studies are needed to explore the underlying mechanisms for these differences and to explore modified dietary treatment options that improve skeletal and cardiac muscles simultaneously.

## Materials and methods

### Ethics

All animal experiments were conducted in accordance with the guidelines for animal experiments at the National Institute of Advanced Industrial Science and Technology (AIST), The University of Tokyo, and international ARRIVE guideline^42^. The experimental protocol was approved by the Institutional Animal Care and Use Committees at AIST (Permission No: 2020-358) and The University of Tokyo (Permission No: P20-012). Animal euthanasia was performed using CO_2_ gas at 30–70% displacement rate of the cage volume/min using a flow meter according to the American Veterinary Medical Association (AVMA) euthanasia guideline^43^. After their respiration ceases, CO_2_ gas flow was maintained for more than 1 min and cardio-respiratory arrest was confirmed.

### DMD rat model

Using a CRISPR/Cas9 gene-editing system, we have established a rat model with an out-of-frame mutation in *DMD* from the Wistar-Imamichi strain^11^. The established DMD rats have mutations in exon3 and exon16 in *DMD* (cDNA sequence; exon3 del, exon16: c.19491950 insT, c.19521953 CG > AT). Animals were maintained in groups of 2–4 per cage at 23 °C under a 12-h light/dark cycle (lights on at 8 AM) and food and water were supplied ad libitum.

### Dietary intervention

Newborn male DMD rats were randomly assigned to 2 groups (n = 6 for each group), those receiving the normal diet (ND) or the medium-chain triglyceride containing ketogenic diet (MCTKD)^22^. Also, their wild-type (WT) littermates fed the ND served as a control group (n = 4). The 36-week dietary intervention period was started at the time of weaning (3 weeks of age) and continued until 9 months of age. The animals were fed 2 types of MCTKD. For the first 10 days, MCTKD with a ratio of fat weight to carbohydrate plus protein weight (ketogenic ratio; KR) of 2.0 was used. For the remainder of the feeding period, MCTKD with a KR of 1.4 was used. The calorie ratios in MCTKD (KR 2.0) derived from fat, carbohydrate, and protein were 82%, 10%, and 8%, respectively. The calorie ratios in MCTKD (KR 1.4) derived from fat, carbohydrate, and protein were 77%, 10%, and 13%, respectively (Supplemental Fig. 1B). The calorie ratios in ND (standardized AIN93M feeding) derived from fat, carbohydrate, and protein were 10%, 77%, and 13%, respectively. The calorie counts of MCTKD (KR 2.0), MCTKD (KR 1.4), and ND were 6.2 kcal/g, 5.9 kcal/g, and 3.9 kcal/g, respectively.

### Electrocardiogram evaluation

Inhalation anesthesia was used during electrocardiogram and echocardiography experiments; DMD rats were anesthetized with 1–3% isoflurane and wild-type rats were anesthetized with 2–4% isoflurane. All rats were placed in a prone position on a heating pad with embedded electrocardiogram leads (THM150, FUJIFILM VisualSonics, Toronto, ON, Canada). Each limb was fixed with tape and electrode gel was applied to the electrocardiogram electrodes. The electrocardiogram was sampled every 0.65 ms with a sampling rate of 8000 Hz. Acquired data were exported to an analysis software (Labscribe4 with electrocardiogram analysis module, iWorx Systems, Dover, NH, USA). The electrocardiogram data were filtered by a bandpass filter with a 15 Hz high-frequency passband and a 200 Hz low-frequency passband and the averaged electrocardiogram was automatically generated from 8 to 10 consecutive waveforms (Fig. 1). Electrocardiogram variables of heart rate, P-wave amplitude, R-wave amplitude, PR interval, QRS duration, and QT interval were automatically measured by the software (n = 6 for ND-fed DMD rats, n = 6 for MCTKD-fed DMD rats, n = 4 for WT rats).

### Echocardiography evaluation

Echocardiographic images were acquired under isoflurane anesthesia using a small-animal digital echocardiography imaging system (Vevo®3100, FUJIFILM VisualSonics, Toronto, ON, Canada). Images of short-axis and long-axis views were recorded at frame rates of 100 fps. The obtained digital raw data were transferred to VevoLAB software and speckle tracking strain analysis was performed using VevoStrain software (FUJIFILM VisualSonics, Canada)^40,41^. From the M-mode short-axis view, LV anterior wall thickness, posterior wall thickness, end-diastolic diameter, and fractional shortening (FS) were calculated. From the LV long-axis view, LV endocardium borders at end-diastole and at end-systole were manually traced and the ejection fraction was calculated using Simpson’s method. Speckle tracking strain analysis variables were also calculated by manual tracing of the LV endocardial and epicardial borders. Peak strain values of all LV segments (strain global), peak strain rate at systole (strain rate S-peak), and peak strain rate at early diastole (strain rate E-peak) from circumferential, radial, longitudinal, and transverse standardized strains and strain rates were calculated (Supplemental Fig. 2). Each echocardiographic value was calculated from the average of 3 consecutive heartbeats (n = 6 for ND-fed DMD rats, n = 6 for MCTKD-fed DMD rats, n = 4 for WT rats).

### Histological analyses

After measuring body weight, the rats were euthanized, and their hearts were weighed. The heart weight ratio was calculated from the whole heart weight divided by their body weight (BW). Paraffin-embedded 4–5 μm thick sections of LV tissue were stained with hematoxylin eosin, Masson’s trichrome, and IgG immunostaining. For IgG immunostaining, the sections were incubated with cardiac troponin T primary antibody (#MA512960, Thermo Fisher Scientific, MA, USA) overnight at 4℃, washed in PBS, and then incubated with 1:100-diluted AlexaFluor-conjugated secondary donkey anti-mouse IgG H&L Alexa Fluor 488 (AB_2340846; Jackson ImmunoResearch Inc, PA, USA) and goat anti-rat IgG H&L Alexa Fluor 647 (ab150159; Abcam, Cambridge, UK) antibodies for 1 h at room temperature. All samples were visualized using a BZ-X810 fluorescence microscope (Keyence, Tokyo). The Masson’s trichrome positive stained area and IgG positive stained area were quantified as described previously^12^ (n = 6 for ND-fed DMD rats, n = 6 for MCTKD-fed DMD rats, n = 4 for WT rats).

### Statistical analysis

All data are presented as the mean ± SEM. Evaluated variables were compared by one-way ANOVA followed by the Tukey–Kramer multiple comparisons test. Results of the comparison of P values of ND-fed DMD rats with MCTKD-fed DMD rats are described in the tables. Differences were considered statistically significant at p < 0.05.

## Supplementary Information

Supplementary Information.

Supplementary Information.

## Abbreviations

DMD: Duchenne muscular dystrophy
FS: Fractional shortening
KD: Ketogenic diet
KR: Ketogenic ratio
LCT: Long-chain triglycerides
LCTKD: Long-chain triglycerides containing ketogenic diet
LV: Left ventricular
MCT: Medium-chain triglycerides
MCTKD: Medium-chain triglycerides containing ketogenic diet
ND: Normal diet
PUFA: Polyunsaturated fatty acid
WT: Wild type
