# Supplementary figures and images for: A medium-chain triglyceride containing ketogenic diet exacerbates cardiomyopathy in a CRISPR/Cas9 gene-edited rat model with Duchenne muscular dystrophy
Source: Sci Rep. 2022 Jul 8;12:11580. doi: 10.1038/s41598-022-15934-9 (PMC9270409; doi:10.1038/s41598-022-15934-9)

Supplemental Figure 1

A

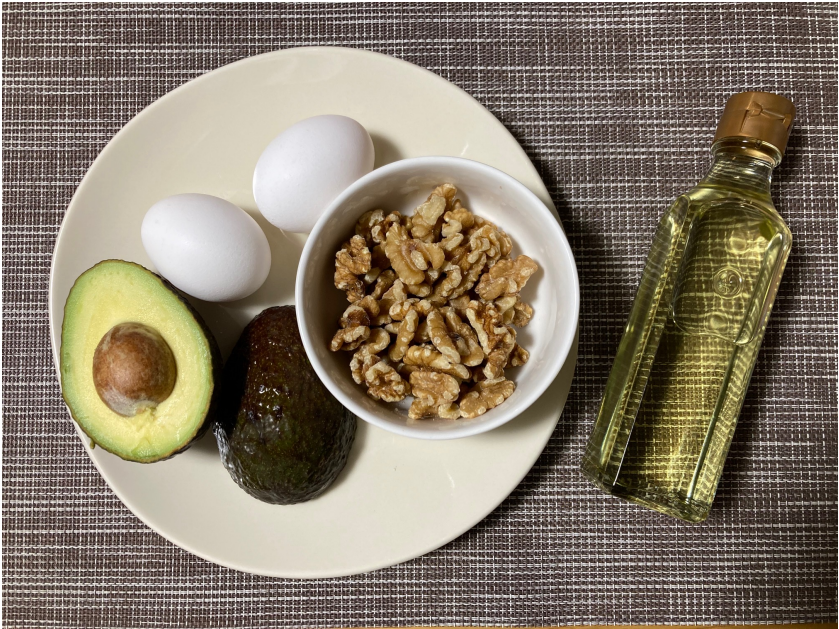

B

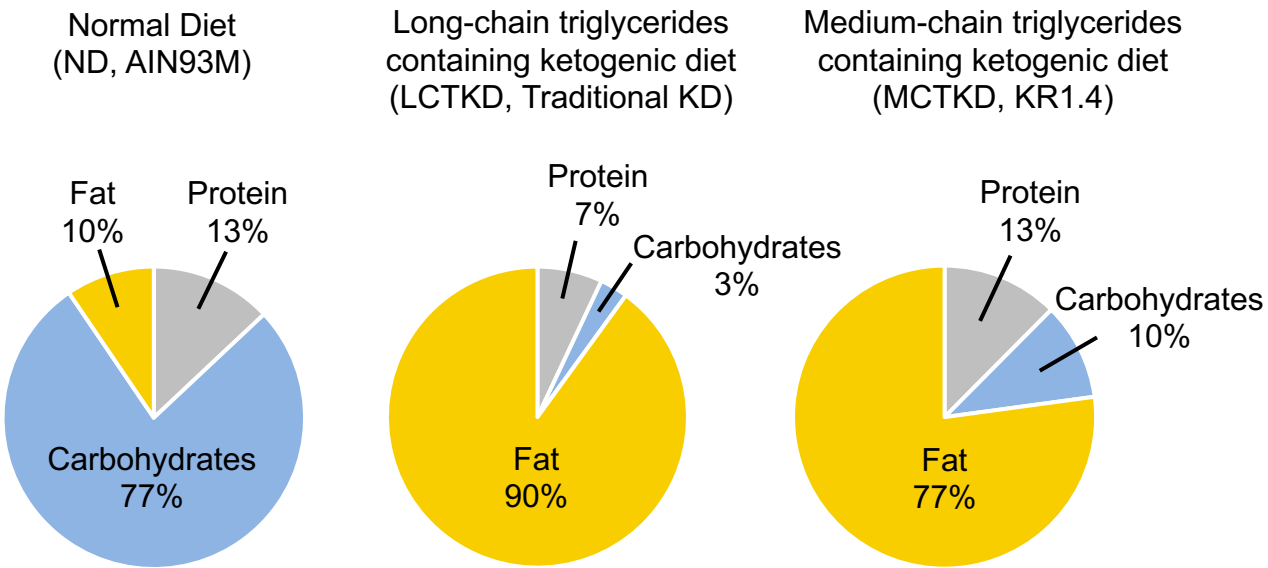

Supplemental Figure 2

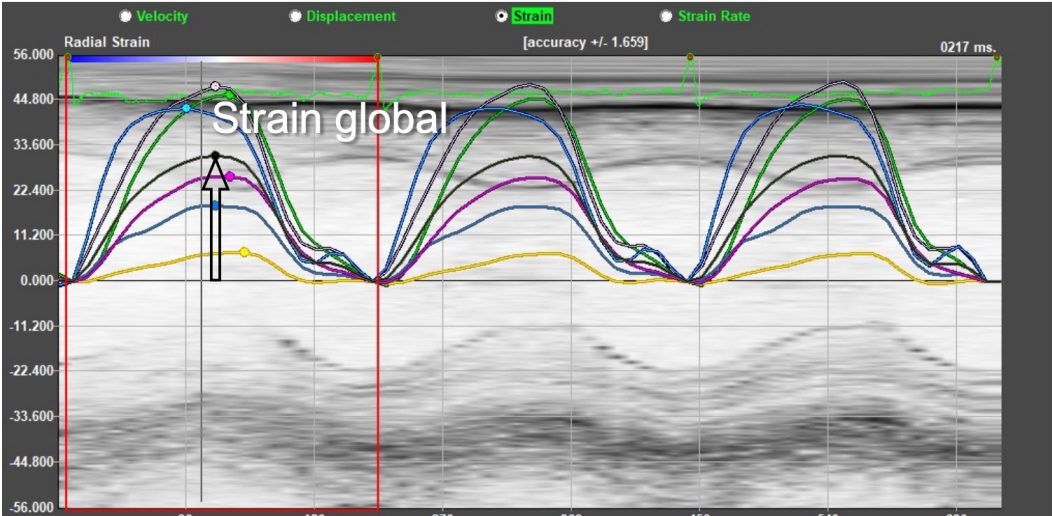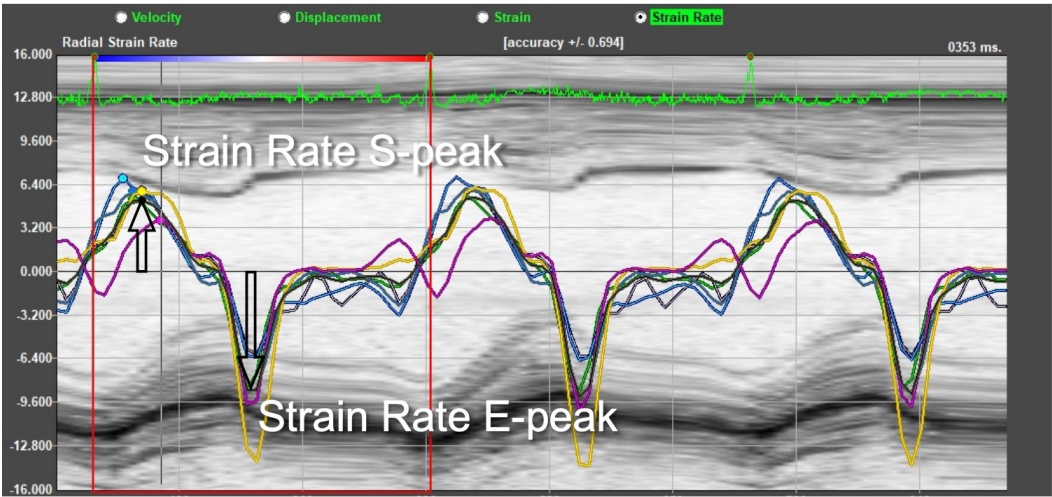

Supplemental Figure 3

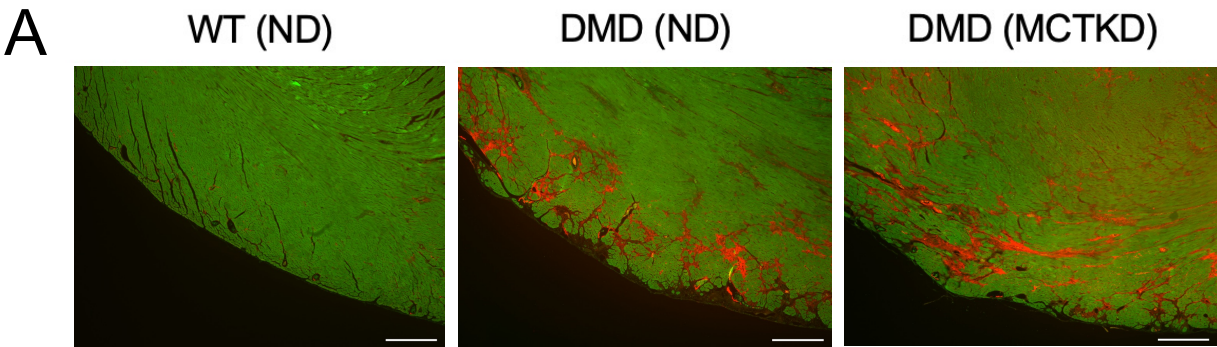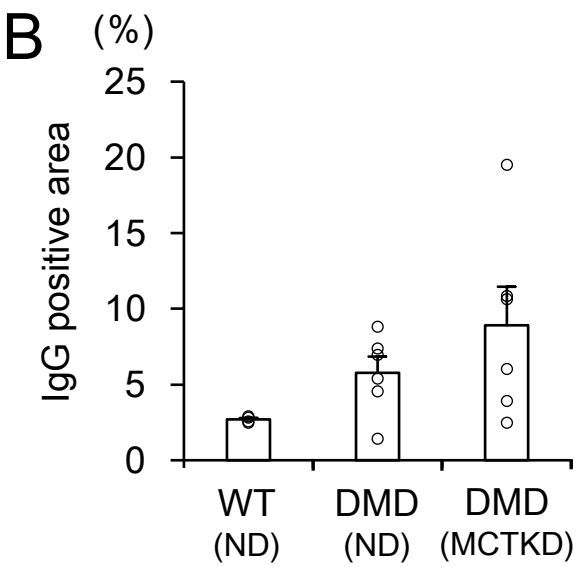

Supplement: Supplementary file 1 — Supplementary Information. [file 41598_2022_15934_MOESM1_ESM.pdf]
