# Supplementary material for: A medium-chain triglyceride containing ketogenic diet exacerbates cardiomyopathy in a CRISPR/Cas9 gene-edited rat model with Duchenne muscular dystrophy
Source: Sci Rep. 2022 Jul 8;12:11580. doi: 10.1038/s41598-022-15934-9 (PMC9270409; doi:10.1038/s41598-022-15934-9)
Supplement: Supplementary file 2 — Supplementary Information. [file 41598_2022_15934_MOESM2_ESM.docx]

***Supplemental Figure 1. Overview of ketogenic diets.***

(A) A representative sample image of a typical ketogenic diet.

(B) Pie charts indicate the percentages of nutritional components in the Normal Diet (ND; AIN93M standardized feeding), long-chain triglycerides containing ketogenic diet (LCTKD; traditional ketogenic diet), and medium-chain triglycerides containing ketogenic diet (MCTKD).

***Supplemental Figure 2. Representative images of speckle tracking strain and strain rate analysis.***

Representative images of radial strain and radial strain rate analysis.

The colored curves indicate strain and strain rate of each segment. The black curve indicates strain and strain rate global of all segments. The values of peak strain at systole (Strain global), peak strain rate at early systole (Strain Rate S-peak), and peak strain rate at early diastole (Strain Rate E-peak) were analyzed (black arrows).

***Supplemental Figure 3. Representative images and quantitative results of IgG immunostaining.***

(A) Representative sections of red-color-stained IgG positive area showing myocardial damage and green-color-stained troponin T showing background cardiomyocytes. Scale bars indicate 500 μm.

(B) The quantitative result of IgG positive area did not reach a significant difference (P = 0.421) between the MCTKD-fed DMD rats (8.9 ± 2.5%) and the ND-fed DMD rats (5.8 ± 1.0%).

Data is expressed as the mean ± SEM and the values were compared by one-way ANOVA followed by the Tukey-Kramer multiple comparisons test (n = 6 for ND-fed DMD rats, n = 6 for MCTKD-fed DMD rats, n = 4 for WT rats).
